# Automation of flow analysis in scleral vessels based on descriptive-associative algorithms

**DOI:** 10.1038/s41598-023-31866-4

**Published:** 2023-03-21

**Authors:** Bekzhan Kerimkhan, Alexander Nedzved, Ainur Zhumadillayeva, Kanagat Dyussekeyev, Gulzhan Uskenbayeva, Bakhyt Sultanova, Leila Rzayeva

**Affiliations:** 1 Faculty of Information Technologies, L.N. Gumilyov Eurasian National University, Astana, 010000 Kazakhstan; 2 Department of Computer Applications and Systems, Belarusian State University, 220004 Minsk, Belarus; 3 Faculty of Innovative Technologies, Karaganda Technical University, Karaganda, 100000 Kazakhstan; 4 Department of Computer Engineering, Astana IT University, Astana, 010000 Kazakhstan

**Keywords:** Engineering, Mathematics and computing

## Abstract

Blood flow reflects the eye's health and is disrupted in many diseases. Many pathological processes take place at the cellular level like as microcirculation of blood in vessels, and the processing of medical images is a difficult recognition task. Existing techniques for measuring blood flow are limited due to the complex assumptions, equipment and calculations requirements. In this paper, we propose a method for determining the blood flow characteristics in eye conjunctiva vessels, such as linear and volumetric blood speed and topological characteristics of the vascular net. The method preprocesses the video to improve the conditions of analysis and then builds an integral optical flow for definition of flow dynamical characteristic of eye vessels. These characteristics make it possible to determine changes in blood flow in eye vessels. We show the efficiency of our method in natural eye vessel scenes. The research provides valuable insights to novices with limited experience in the diagnosis and can serve as a valuable tool for experienced medical professionals.

## Introduction

The change in the blood flow rate in vessels reflects the health state and level of disease. When microcirculation is disturbed in the retina due to diseases or conjunctiva of the eye, microhemodynamic disturbances in the brain, myocardium, and other organs happen. That connected with changes of vascular net and characteristics of blood. The change in the blood flow rate in the blood vessels of the eye reflects the change in physiological state. Thus, one can indirectly judge the state of blood flow in other organs and tissues and reveal its disturbances at the early stages of the vascular pathology development by checking the size of the vessels and the parameters of hemodynamics in the eye's tissues. In this regard, the search for non-invasive methods for studying the state of blood vessels and hemodynamics can improve the early diagnosis of cardiovascular diseases.

The detection of non-invasive methods for determining of the dynamical state of blood vessels is a very important task. Using a non-invasive method for determining the state of blood vessels and blood flow is important in developing modern, effective diagnostic methods. Non-invasive observation allows to research of optical characteristics of the retina and other fundus structures' vascular system. Changes in blood vessels and blood flow allow estimating an integral characteristic of the body's state. Changes in blood vessels are visible by fundus lens. They are connected with integral characteristic of the state of the body as a whole and the state of the visual system, in particular. Changes in the ratio of the diameter of arteries, veins, and blood vessels, increased curvature of blood vessels, and other changes are early signs of retinal vascular damage caused by diseases. Changes in blood flow correlate with the changes in blood flow in the microvasculature of the brain, heart, and kidneys. Therefore, one can obtain important information about microcirculatory blood flow and its disorders in atherosclerosis, arterial hypertension, diabetes mellitus, and other diseases by measuring the linear blood flow velocity.

Blood flow monitoring is very famous problem. There are many methods of such flow researching, for example Doppler ultrasonography and velocimetry, laser Doppler flowmetry, and dedicated handheld devices. Blood flow parameters are determined only for straight sections of the microvascular net by such methods. Changes in the nodes, branches, and complex fragments of the vascular network have not been studied in academic research, to the best of our knowledge. It is connected with complexity of the interpretation of the results of motion measurement.

Almost all modern methods of this direction include interactive methods and carry out the analysis of point structures. The blood flow rate is one of the most critical parameters which characterize the body's functionality of the circulatory system. Velocity characteristics of blood flow directly connected with many significant characteristics such as blood pressure, human age, frequency of contractions of the heart muscle, quality characteristics of blood and etc. The blood flow rate plays a considerable role in diagnosing heart and vascular diseases and monitoring the state of the whole body. Today there are many portable devices (fitness bracelets, holders) for blood flow monitoring. However, the interpretation of the results is not always adequate. This is because most analyses do not consider the features of the changes in processes that occur in the nodes and complex fragments of the vasculature. Methods of analyzing and processing images allow for tracing such changes. In this case it is very important to take into account the description of dynamical processes in the nodes and complex fragments of the vasculature. The descriptive methods allow defining computer vision algorithm that is based dynamical process analysis in all vessels network.

There are many algorithms for processing and analysis of vascular medical image. Several reviews are described them. In 2003, in report for the NHS (Health Technology Assessment), Sharp published an overview about digital analysis of vessel. The basic task of such research was a digital analysis of different technologies for processing and analyzing diagnostic images. It failed because digital technologies were only at an early stage of their development in this field^1^. The first reviews of algorithms of vascular image analysis for automation of medical diagnostics can be described in^2,3^. Then a comparative overview of algorithms was published by Kirbas and Quek^4^ where isolating vessels and elongated objects was detected on two-dimensional and three-dimensional space for different medical problems. The vessels segmentation algorithms have many ways of realization from time of fist fundus camera but a quality review of segmentation and registration of the retina vessels was presented by Mabrouk et al.^5^. In this paper only tasks of boundaries and detection central lines (skeleton) of the vessels was described. There are complex solutions for vessels analysis, for example in paper^6,7^ the automatic diagnosis of diabetic retinopathy was analyzed for retinal images by algorithms of computer vision. But the review^7^ is unique because it collected papers with different algorithms for automation analysis of vessels on medical images for diagnostics in diabetic retinopathy published between 1998 and 2008. Most interest have algorithms of vessels segmentation on color images received for fundus cameras. For segmentation, it includes algorithm that based on methods of thresholding and region growing. The next review^8^ have description of algorithms only for isolation of vessels that try to use topological description for algorithms.

Recently, machine learning methods^9,10^ and neural networks^11,12^ have been actively used to study retina vessels. Almost all papers describe vessel segmentation problems on a static image.

Analysis of this topic allows us to determine trends and problems in digital vascular processing. All articles described above spend researching of static retina images in two-dimensional space. It is very complex to determine the optimal algorithm for each stage of vessel image processing. In many cases, some methods have some defects and troubles. Some articles develop and improve approaches based on the existing ones. Abramoff et al.^13^ 's application of an automated system is not recommended for clinical use to detect vascular pathology based on existing algorithms. On the other side, modern equipment makes it possible to study dynamic changes in blood vessels, and this direction is beginning to develop actively^14^.

This work aims to automate the research of eye diseases on base analysis of the blood flow velocity in the vessels for diagnosing diseases of the respiratory and cardiovascular systems.

## Materials and methods

### Acquisition of sclera vessels video sequence

Vascular examination is usually carried out using a fundus lens. Fundus lenses are designed for wide-field stereoscopic examination of the fundus by biomicro-ophthalmoscopy, as well as for laser interventions on the inner membranes and structures of the eye. Due to the large field of view of fundus lenses, they allow obtaining a panoramic image of a significant part of the fundus without requiring additional manipulations with the lens itself. Fundus lenses in combination with a slit lamp binocular microscope make it possible to carry out a consistent view of the fundus by scanning the light slit, as well as to examine the retina in detail. Due to the high resolution, stereoscopicity and image quality, fundus lenses make it possible not only to get an approximate idea of the pathology, but also morphological detailing of the changes found, their exact spatial-depth localization. A clear image makes it possible to diagnose even minor abnormalities in the vitreous humor and in the fundus.

In our case for measuring the morphometric parameters of the vessels of the bulbar conjunctiva of the eye, we use ophthalmology complex which consists of a personal computer, a monochrome camera Imper × 1023 Bobcat IGV-B1410M, a laser device for aiming and focusing the camera on the vessels of the bulbar conjunctiva and a device for synchronizing pulsed illumination with the construction of video frames^15^, having an adjustable frequency and brightness of light. On the basis of this equipment, a sequence of images of the scleral vessels is formed as video where it is possible to monitor changes in the vessels and diagnose pathological processes.

**Figure 1:**
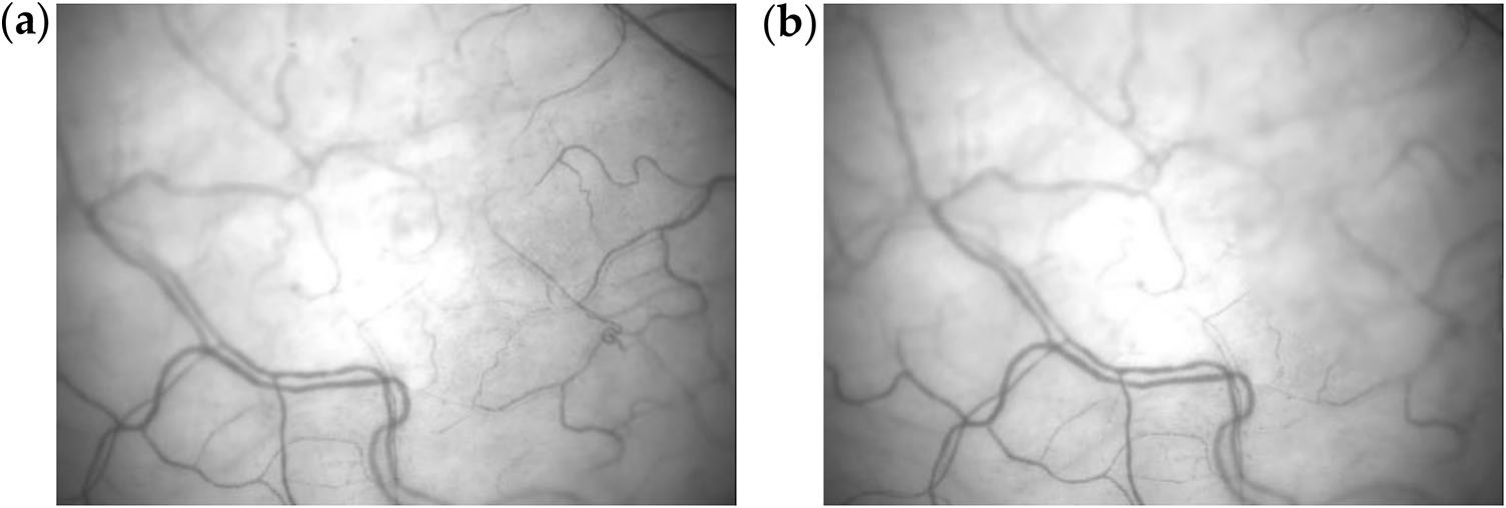
Two adjacent images in a video sequence: (**a**) The first frame; (**b**) The first frame. In image vessels shifted, a right fragment of image has a less contrast, a bright spot in the center illuminates other objects.

We use gray scale video. However, this video sequence has a number of disadvantages that make automatic analysis difficult. During acquisition, it is impossible to fix the eye, as a result, the field of view is always moving and the brightness is randomly changing. for example, a region with vessels can move to other place of headlights and the sharpness of its fragments can change as Fig. 1. There are cases when the image completely jumps to another position or image contrast disappears. As a result, the object on which the observation is carried out disappears. This leads to difficulties during the analysis and requires additional processing of video-sequence.

### Problems of processing and analysis video

The video turns out to be unstable and requires division into fragments that can be stabilized. Such way procedure of vessels analysis has many troubles. More important troubles look like this:

- the heterogeneity of contrast in the image requires additional clarification of the region of interest for processing by estimation of the information content in area of objects;
- loss of sharpness leads to the need to cut out a fragment of a bad image;
- strong jumps when preparing a video requires splitting a sequence of images into fragments in which the same object is present;
- movement object lead to additional stabilization of every fragment of video.

In result, analysis of dynamical characteristics of blood flow require to definition solution of next problems:

- extracting quality fragment of video by definition regions of interest and the cropping of regions with maximum information content;
- stabilization of such video-fragment;
- vessels masking;
- calculation of blood flow characteristics.

This article solves these troubles and problems by organization of cascade of computer vision methods and algorithms.

### Definition regions of interest

The video with a fundus lens is not stable, images constantly change the sharpness and position of the object. Therefore, one of the most important tasks is to determine the region of interest (ROI) where the object has good contrast. Contrast measures the relative decrease in the luminance in an image. It can be defined locally or globally. The local contrast can be estimated depending on the local differences in gray levels of image fragment. It is possible to define a contrast as a function of image edges.

There is task of definition of region with high level of information content. The informativeness of images should be understood as the total amount of information obtained by spatial characteristics during their perception or analysis. A more accurate assessment of the information content of an image appears to be complex and dependent on several indicators, including local contrasts and detail. The simplest method from this group is based on the extraction of low and high frequencies of images with their subsequent summation. However, the images of blood vessels are a set of narrow extended objects, therefore, to determine the information content, high frequencies are sufficient.

The most striking indicator of information content in the image is high gradient levels. A gradient is a vector quantity that shows the direction of the steepest increase in a certain quantity:

1 $$\overline{g} = grad\,g=\left(\frac{\partial I}{\partial x},\frac{\partial I}{\partial y}\right),$$

where $I$ is image, $x$,$y$ are horizontal and vertical coordinates.

**Figure 2:**
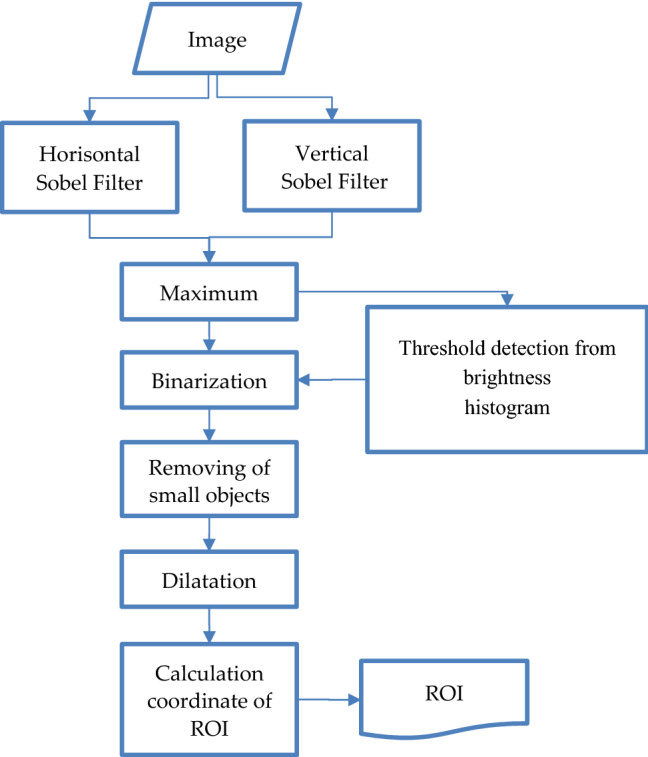
The process of ROI definition for region with good contrast.

The quick and convenient way to obtain high-frequency components is to use Sobel filters. The Sobel operator is a discrete differential operator that calculates the approximate value of the image brightness gradient. There are two type of Sobel operator for vertical and horizontal gradient. Maximum from it allow to collect results from every direction. The next trouble is definition of gradient level where image has good contrast. For solution of such problem in common scheme of processing we create addition block for definition of threshold for gradient like as Fig. 2.

**Figure 3:**
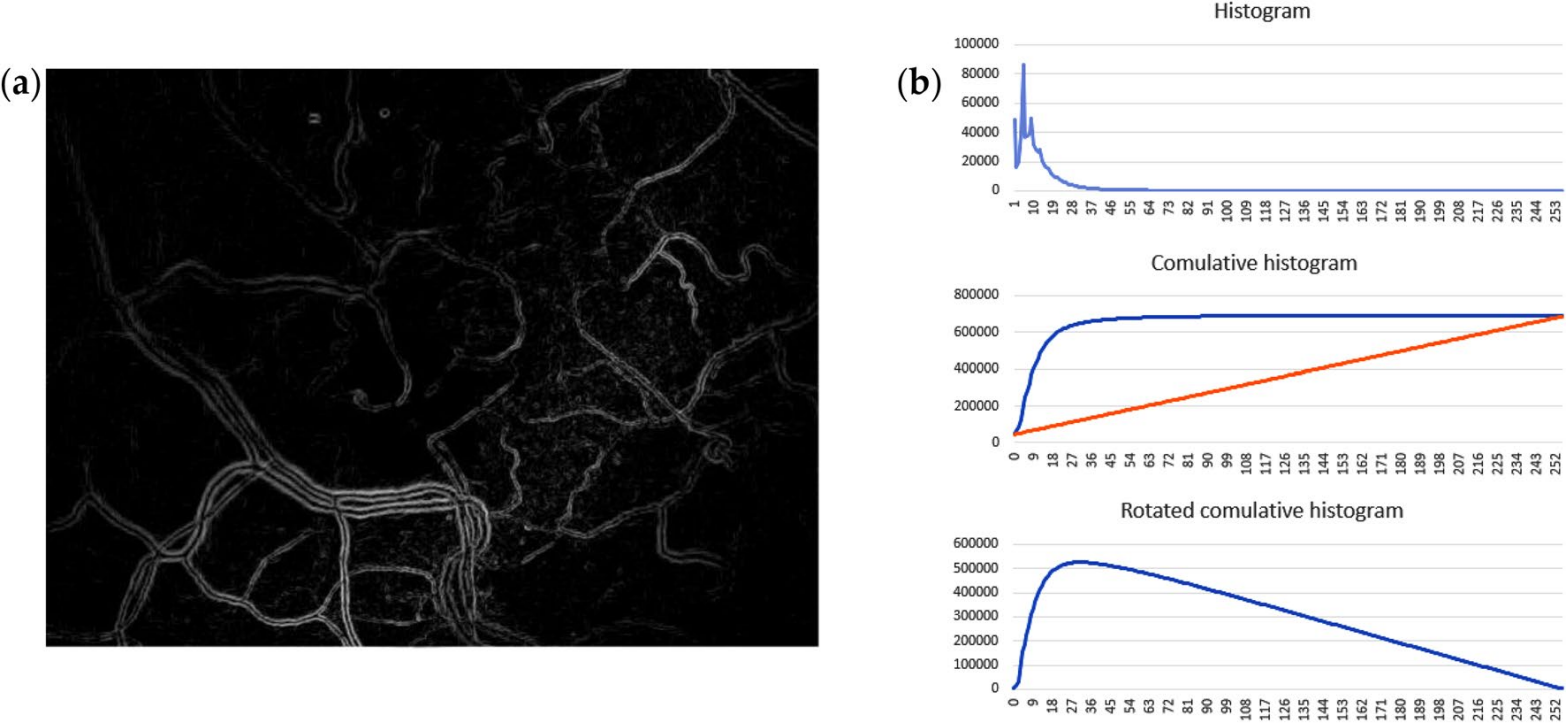
Characteristics of gradient image: (**a**) gradient image as maximum from vertical and horizontal Sobel component; (**b**) set of histograms of gradient: classic, cumulative with line between border points, rotated cumulative histogram.

The procedure od threshold detection consist of procedure of brightness histogram estimation. Maximum from every component of gradient. The informativity are calculated as contrast estimation^16^:

2 $$C = \frac{{I}_{max}-{I}_{min}}{{I}_{max}+{I}_{min}},$$

where ${I}_{max}$ and ${I}_{min}$ is maximum and minimum value of brightness on gradient image. This estimation allows to define case with very low informativity if $C<0.3$. Then it is necessary detect threshold for vessel binarization. For such operation we use cumulative histogram that demonstrate on Fig. 3.

The most informative fragment of the gradient image is characterized as very bright. Nevertheless, it is very difficult to determine the position of the threshold for brightness on the histogram in the classical representation. In order to facilitate the interpretation of the histogram, It is translated into a cumulative form where it correspond to the integral representation of the frequencies at each brightness level as on Fig. 3b. The optimal position of the threshold for brightness will correspond to the point of maximum bend of this histogram. if a curve of cumulative histogram turns along the line between the border points, threshold of brightness is detected as the point with maximum level. After binarization by this threshold, a lot of small debris remains at the image. they are removed by size. In addition, an additional operation of dilation is used to linking of the separated objects.

For coordinates detection, contours are created for all objects in the image by which the maximum and minimum positions are determined. Coordinates of this positions correspond to bounding box of ROI with maximum information content. After processing all video-sequence the set of bounding box coordinates are created.

### The cropping of regions with maximum information content

The information-rich region of the image contains a qualitative fragment of the vessels, which can be tracked on the other frames of the video sequence. These fragments of the image can be watched video only during a certain period of time. The presence of a vessel area on the frames of the video sequence can be tracked based on the movement of the ROI and the center of mass of the contrast, which is calculated as

$${x}_{c} = \frac{\sum_{i}{x}_{i}\cdot {I}_{i}p}{n},$$

3 $${y}_{c} = \frac{\sum_{i}{y}_{i}\cdot{I}_{i}p}{n}$$

where ${x}_{c}$ and ${y}_{c}$ is coordinate of center of mass, $i$ is sets of border information points, ${I}_{i}$ is intensity pixel (${x}_{i}$*,* ${y}_{i}$) with high informatively, *p* is normalization coefficient that aligns maxim intensity of image fragment inside ROI to 100% weight.

Control of center of mass position allow to detect time fragment on video that include ROI with vessels. These coordinates are controlled for every frame. If shifting of center of mass more than constant value the frame marked like as last in sequence of image with contrast vessels. This constant value is defined by u (in our case this constant is equal 100). In parallel ROI of every frame is corrected by comparison with common coordinates for all frames of contrast vessels fragments:

$${(x}_{c1}{y}_{c1}) =(\mathrm{min}(\underset{f,j}{\bigvee }{x}_{f,j}),\mathrm{min}(\underset{f,j}{\bigvee }{y}_{f,j}))),$$

$${(x}_{c2}{y}_{c2}) =(\mathrm{max}(\underset{f,j}{\bigvee }{x}_{f,j}),\mathrm{max}(\underset{f,j}{\bigvee }{y}_{f,j}))),$$

$${w}_{b} =\mathrm{min}(\mathrm{max}(\underset{f,j}{\bigvee }{x}_{f,j})-\mathrm{min}(\underset{f,j}{\bigvee }{x}_{f,j})),$$

4 $${h}_{b} =\mathrm{min}(\mathrm{max}(\underset{f,j}{\bigvee }{y}_{f,j})-\mathrm{min}(\underset{f,j}{\bigvee }{y}_{f,j})),$$

where ${(x}_{c1}{y}_{c1})$ and ${(x}_{c2}{y}_{c2})$ is corner points of bounding box for all frame, (${w}_{b}$, ${h}_{b}$) is width and height of common small box for all frame, *j* is index in ROI of frame image, *f* is index of frame.

As result after missing of center every image is cropped by bounding box and we have sequence of cropped image with common contrast fragment of vessels, coordinates of centers of contrast ROI for every frame and common box of contrast regions around those centers.

### Creation and stabilization fragments of vessels video

After the previous actions, a set of videos has been created. Now, it is necessary to build a fixed coordinate system for each video from set for possibility of motion estimation into vessels.

The current video contains a change in the coordinates of the object, which includes the movement of the camera and the object itself. As the camera moves, different objects can get the same coordinates, even if the objects were static. It is necessary:

- determine the origin of coordinates;
- compare two consecutive frames with each other;
- find a transformation that will translate object coordinates on the current frame into coordinates relative to the origin, taking into account all camera movements.

**Figure 4:**
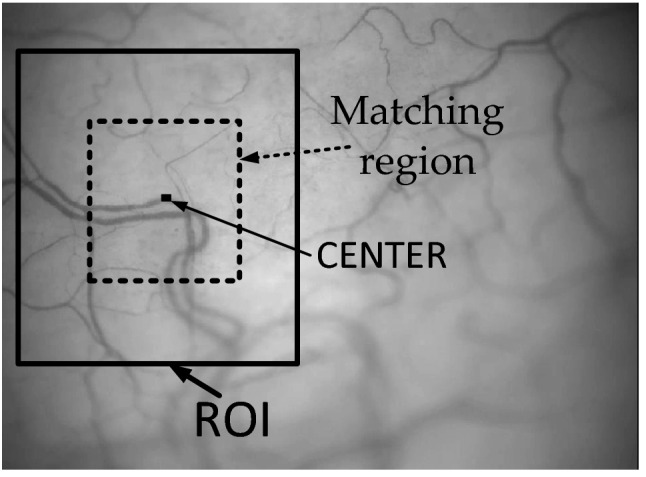
Definitions of ROI and matching region.

The using of frame stabilization leads to a reduction of the images size, because It is necessary to cut a common fragment from every image of video-sequence. Usually, the upper left corner of the first frame is taken as the origin. But the video of the vessels is characterized by the random movement and the position of the first frame does not always correspond to a good position. In previous part of this article we define ROI of contrast fragment and its position. It is possible to use such information and try to stabilize video by fragment into ROI. We use only 50% of this ROI with the same position of center. For origin of coordinates we define such center from ROI of first frame as Fig. 4.

The next step of frame comparation allow to take different result for different algorithms of image comparation. We try at least tree types of such algorithms:

- by methods of machine learning^17^;
- by classical correlation^18^;
- by comparation of key points positions.

All algorithms of machine learning including algorithms on base Kalman filter show bad results. They led to displacement of the vessels from frame to frame.

For images of the vasculature, the comparison method based on classical correlation gives the best results, since it relies directly on the images of the vessels, and not on their features. Image correlation is performed for each frame.

Correlation of images is comparing all possible pixel pairs and generation of map of likelihood that both pixels will have the close value as a function of the distance and direction of separation. In a more mathematical definition, correlation is the convolution of a function of two image. For a digital image *I* of size *M* × *N* and image *J* of size *K* × *L* correlation can be calculated by

5 $${G}_{IJ}\left(k,l\right)= \frac{{\sum }_{m}{\sum }_{n}\left(I\left(m+k,n+l\right)-\overline{I }\right)\left(J\left(m,n\right)-\overline{J }\right)}{\sqrt{{\sum }_{m}{\sum }_{n}{\left(I\left(m,n\right)-\overline{I }\right)}^{2}{\sum }_{m}{\sum }_{n}{\left(J\left(m,n\right)-\overline{J }\right)}^{2}}},$$

where $I(m,n)$ is the pixel intensity value at coordinates $(m,n)$ in the first frame, $\overline{J }(m,n)$ is the intensity value at a coordinates $(m,n)$ in the second frame, $\overline{I }$ and $\overline{J }$ are mean values of the intensity in $I$ and $J$ images respectively. As result the map of correlation is constructed where maximum correspond of point of best crossing. The coordinate shift is calculated as difference of coordinates of the maximum on the correlation image and center of ROI at first frame.

Unfortunately, this way is too long. In practical applications, the correlation array is usually computed using Fourier-transform methods, since the fast Fourier transform^19^ is a much faster method than directly computing the correlation. But it allows to using additional libraries and increase code of software.

Fast and robust image matching is a going by key points comparation. We compare the performance of three different image matching techniques as SIFT^20^, SURF^21^, and Shi-Tomasi Corner Detector^22^ for different image with vessels. For this purpose, the matching evaluation parameters such as the number of key points in images, the matching rate, and the execution time are estimated for every algorithm (Table 1).

**Table 1:** Sample of comparation fragments from two frames by different algorithms.

Base of algorithm	Time (s)	Key points of frame 1	Key points of frame 2	Matches	Match rate (%)
Correlation	0.89	–	–	1	100
SURF	0.13	128	119	96	77,7
SIFT	0.4	62	66	50	78,15
Shi-Tomasi	0.3	22	22	22	100

The matching rate was calculated as:

6 $$Mr = \frac{M*200}{\left(Kp1+Kp2\right)},$$

where $Mr$ is Matching rate characteristics, $Kp1$ and $Kp2$ are counts of detected key points for first and second frames, *M* is count of matched points.

As result the algorithm on base Shi-Tomasi Corner Detector is the fastest algorithm while correlation shows the best quality. SIFT and SURF performs with errors because detect points where vessels change direction. In this time algorithm on base Shi-Tomasi Corner Detector use only points in nodes of vessels branch. This property allows to take best results for algorithm on base Shi-Tomasi Corner Detector on vessels images.

In this way sets key points that characterized by maximum of matching in sequence of images. Sequential analysis of moving points is not efficient. For control of shifting of key points we use optical flow. The best solution of it is Lucas–Kanade algorithm^23^. It is a widely used differential method for points shifting. This algorithm based on the essentially constant flow for a local neighborhood of every pixel. It defines the optical flow equations by the least square’s criterion.

All the neighborhood points have the same motion. Shifting characteristics (${f}_{x}$*,*${f}_{y}$*,*${f}_{t}$)—vertical, horizontal and time^24^ are calculated for these points. The final solution includes two equation and two unknown parameters (7).

7 $$\left[\begin{array}{c}{\varvec{u}}\\ {\varvec{v}}\end{array}\right] = {\left[\begin{array}{cc}{\sum }_{i}{f}_{x\left(i\right)}^{2}& {\sum }_{i}{f}_{x(i)}{f}_{y(i)}\\ {\sum }_{i}{f}_{x(i)}{f}_{y(i)}& {\sum }_{i}{f}_{y\left(i\right)}^{2}\end{array}\right]}^{-1}\left[\begin{array}{c}-{\sum }_{i}{f}_{x(i)}{f}_{t(i)}\\ -{\sum }_{i}{f}_{y(i)}{f}_{t(i)}\end{array}\right],$$

where ***u*** and ***v*** of horizontal and vertical component of motion vectors for key points, *i* is index of pixel in image. The set of such vectors is really good for small motions, but it is not good for a large motion. To deal with this we use pyramids. From bottom to top characteristics of motion are change, small offset vectors are removed and large offset vectors become to small ones. The application of Lucas-Kanade algorithm allow to get optical flow by considering scaling. The algorithm use scale pyramids for solve problem of motion scale. However, there are distance limits for offset points. The vectors ***u*** and ***v*** have good corresponding of shifting only for defined range of distance. The step of image cropping guaranteed to such conditions.

The optical flow allows to construct set of offset key points between two frames in video-sequence. It is possible to use this set for definition offset of frame as median. It will to correspond of most strong shifting. Now we have ROI of contrast region, offset and coordinate for every frame. On base such information video constructed with stabilized position.

### Vessels masking

**Figure 5:**
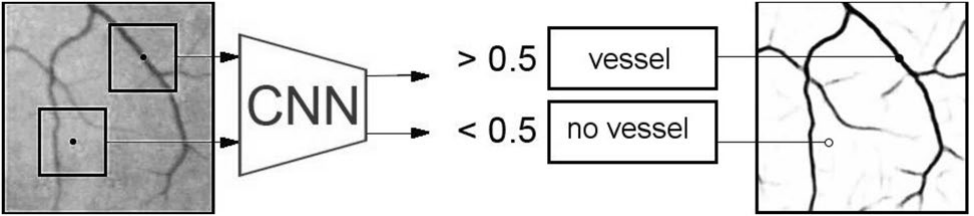
The common scheme of pipeline of vessel segmentation.

Even though the video is stabilized, it still contains a lot of useless information that interferes with motion analysis. Masking of vessels allow to extract important motion in them. The vessel mask is constructed by segmentation. The solution of the problem of segmentation of the vascular net is performed as a classification problem. The learning the convolutional neuron network (CNN) with a sliding window allow to solve it and define vessel class as region on image. The prediction of the label of a class for each pixel is going by analysis pattern in neighbourhood region as in article^25,26^. This small region is used as a source data (Fig. 5).

The fully connected convolutional neural network on base U-net model was used for vascular image segmentation. Such model is supplemented with additin layers. In them Union operators are changed to operators of discretization. In this way, the resolution of the output layer is increased. It allow to combine features of higher resolution from a narrowing region to fragment with an expanding laers. The training of convolutional neural network with such modification is going to more accurate result at the output.

A set of 130 Gy-scale images of eye sclera was collected in Belarussian State Medical University by fundus camera of GigE type Full HD (1920 × 1080) video resolution. each image was divided into fragments with size 352 × 352 pixels. Also we use open source image sets: DRIVE, STARE, CHASE DB1, HRF It was used to train the neural network^27^. In addition, we use pretraining part of the U-net like-SNA architecture^28^ that was proposed in isbi 2012 EM Segmentation Challenge (Segment Neural Membranes).

For improving result of training of CNN a geometric augmentation was used. It use simple geometric transformation: 1) flips, 2) turns, 3) reflections, 4) elastic deformations, and 5) scaling. As result dataset is increased to 650 synthetic images. The NVIDIA GeForce GTX 1080 Ti was used for training process. The training lasted for 700 epochs and batch equal 8.

**Figure 6:**
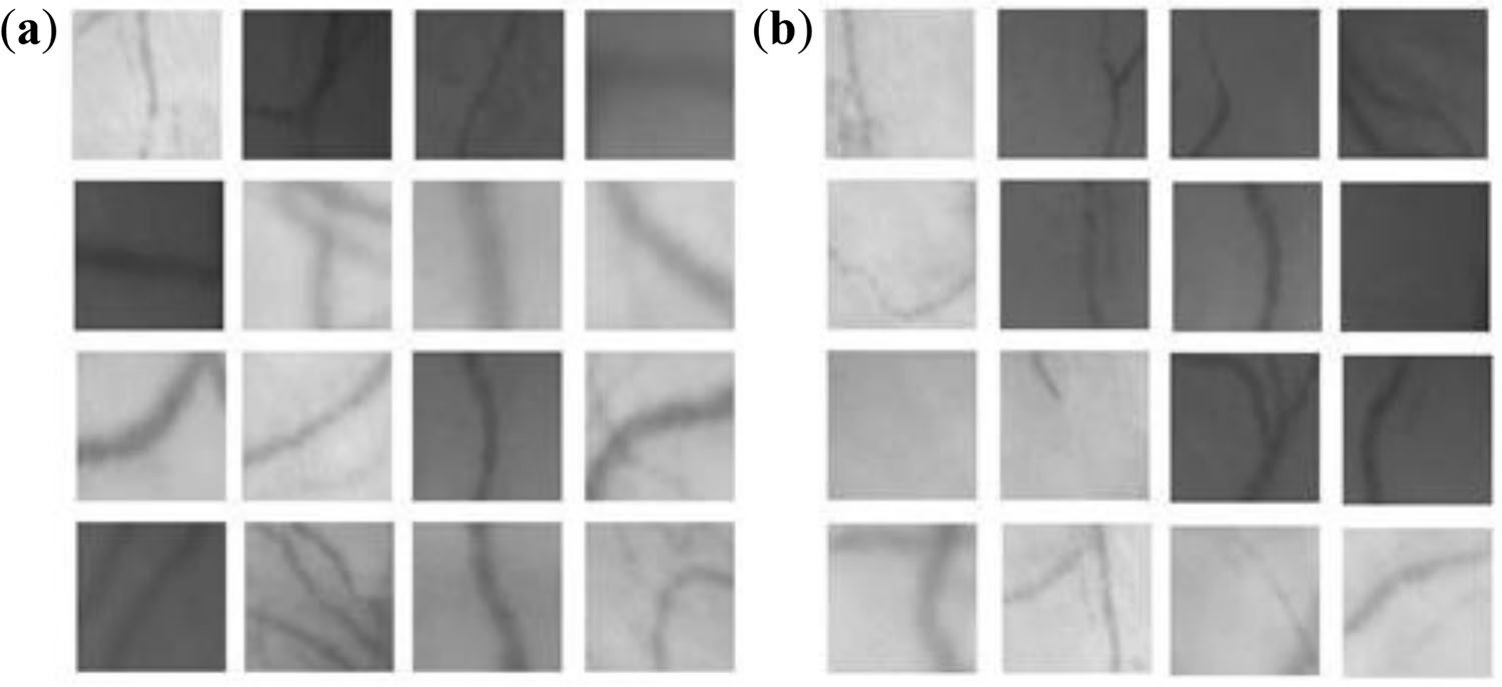
Training patterns: **(a)** with vessel in the center (class 0); **(b)** without vessel in the center (class 1).

For classification is used two classes. One class include regions with vessel in the center and second class collect regions without vessel in the center. Every region has the same size and is selected from the input sample images as Fig. 6. A stochastic gradient descent was used as optimization method. The result of image processing by CNN is the probability for each vessel. It is change from 0 to 1, where 0 stands for vessel, 1 stand for non-vessel and look like image segmentation.

This neural network includes 23 layers of convolution. After it constructed 64-component vector. But we use only two classes. Therefore, convolutions of 1 × 1 size was used on the last layer. The size of the input image is determined by subsampling (2 × 2 max pooling). It allows to guaranty even values of height and width of layer.

**Figure 7:**
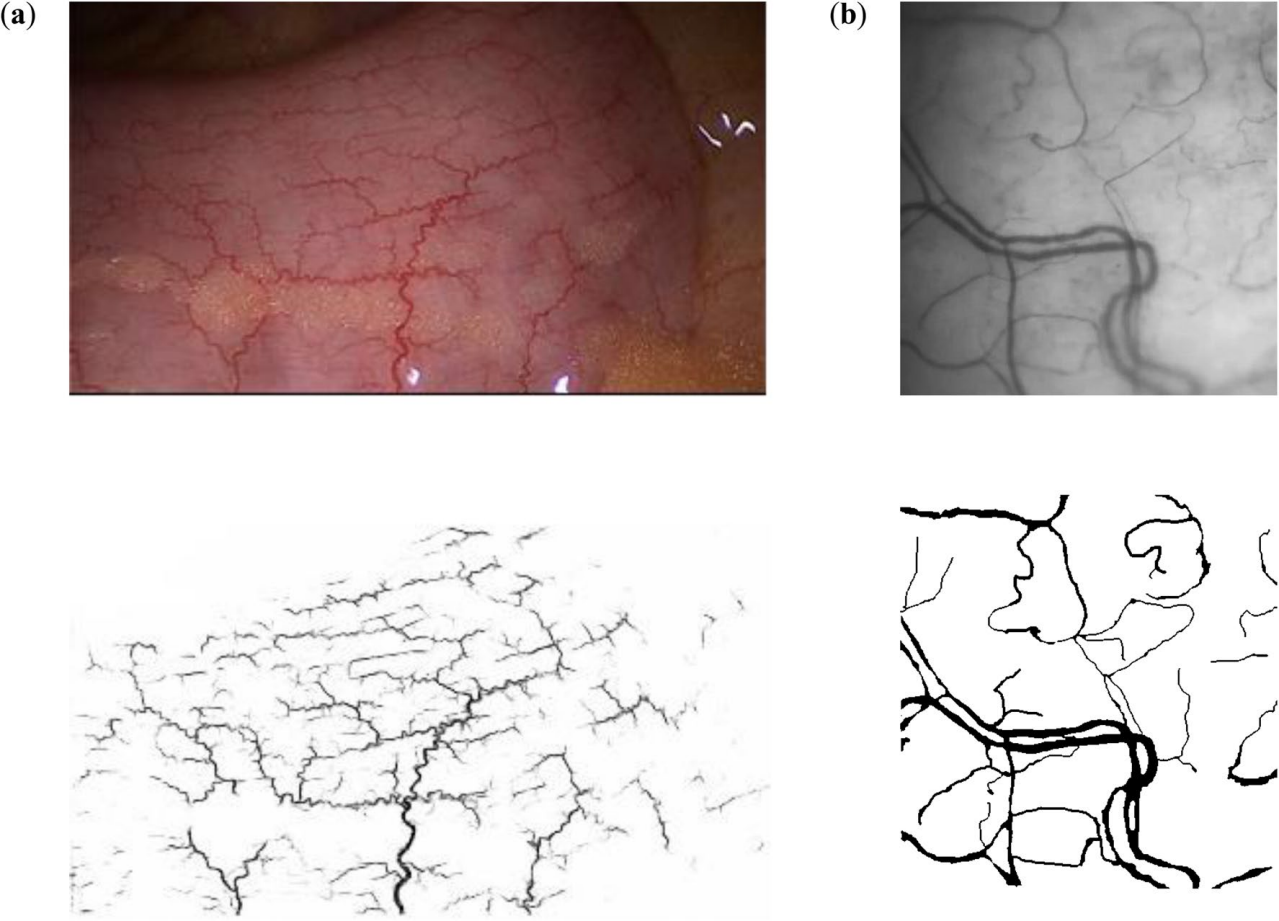
The results of segmentation of the vessels branch and result of segmentation by CNN for different type of image: **(a)** on the endoscopic image; **(b)** image of the fundus.

A concatenation with the corresponding set of features from the narrowing region, and two 3 × 3 convolutions was used in this network. After every layer is going a transformation through the ReLU activation function. This model is going to good results for the segmentation of blood vessels like as Fig. 7.

The standard deviation and accuracy mark were used for estimation of effectiveness of this algorithm^5^. Standard deviation is calculated as:

8 $$\sigma = \sqrt{\frac{1}{n}{\sum }_{i}\left({x}_{i}-\overline{x }\right)},$$

where $n$ is pixel number, ${x}_{i}$ is result label (0 or 1), $\overline{x }$—probability result. Accuracy is normalized number of true answers. Estimation of segmentation effectiveness is shown in Table 2.

**Table 2:** Estimation of segmentation quality for vascular net.

Data	Accuracy	SD
Vascular net detection	0.9517	0.1897
Clear regions	0.9321	0.2135
Common mean estimation	0.9418	0.2016

This segmentation algorithm extract regions of vascular net from gray-scale image with high quality and allow to use this result as mask for vessel analysis.

### Calculation of blood flow characteristics

Before measurement brightness stabilization are spend after masking of stabilized video. It is allowed to normalize densitometric characteristics of images and remove brightness distortion. For realization of it we use specific technics of masking and Anbarjafari algorithm^2^. Every frame in video is change to image where background under mask is reduced to a constant value of the corresponding average brightness around the vessel. Brightness values inside the vessel are stored. In result we have permanent background.

Then Anbarjafari algorithm aligns brightness in video. It use an iterative n th root and n th power color equalization for single generic images. The intensity value of an image is passed through a non-linear transfer function $(x)=\mathrm{ln}(0.5)/\mathrm{ln}(x)$, where $x$ is the image’s mean intensity. The operation is repeated until the final image achieves a mean intensity equal to $\gamma$, set typically to $\gamma =0.5$. The evolution of this method is described in^3^.

As rule boundary effects influence to the analysis of a blood flow. For removing this effect, it is necessary to accurate define region for analysis. Middle line of a vessel corresponds to such region. It can detect by thinning operation. The stabilization video and brightness can be masking such middle line for analysis. Inside this region point of vessels are defined for analysis and are used for calculation optical flow. It allows to estimate dynamical characteristics of blood flow speed that is determinate directly through vessel in vascular net.

This operation allows to decrease extern influence for calculation of velocity characteristics and calculate the instantaneous linear speed in the center of the vessel.

For analysis velocity characteristics in every point of image the map of dense of optical flow is generated from two frame from video. For this solution the algorithm of Gunnar FarneBack technique^24^ show best result. In frame of this algorithm vectors in map of optical flow correspond to the pattern of apparent motion of objects as the motion of objects between every two consecutive frames from video. It is defined by the movement of the object being captured.

**Figure 8:**
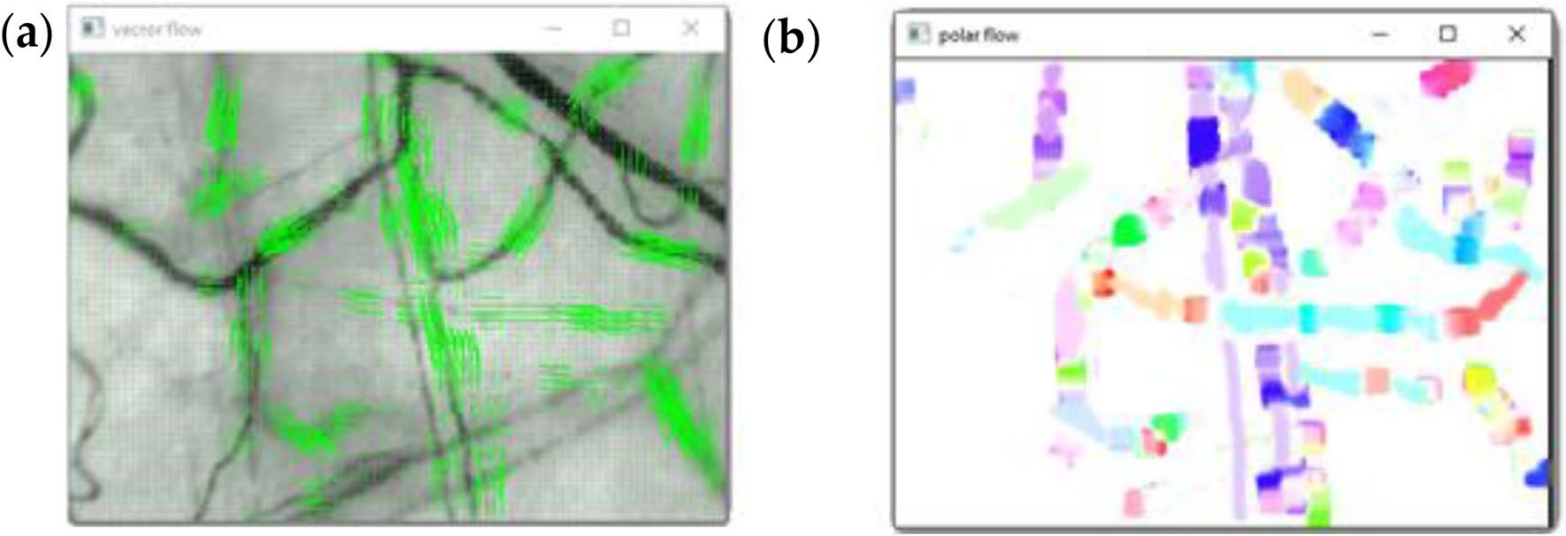
Maps of optical flow for vascular network: **(a)** vector field representation in horizontal/vertical coordinate system, that is traditional for image; **(b)** vector field representation in polar coordinate system.

The FarneBack algorithm destined for dense vector field of optical flow. This algorithm check at all of the points on the image. The changes of pixel intensity are registrated between the two frames, unlike Lucas Kanade which works only on corner points detected by Shi-Tomasi algorithm^22^. For best interpretation horizontal and vertical components of optical flow transform to polar coordinate system as magnitude and phase of vector and represented in HSV color coordinate system where magnitude correspond to brightness and phase is hue, like as Fig. 8.

**Figure 9:**
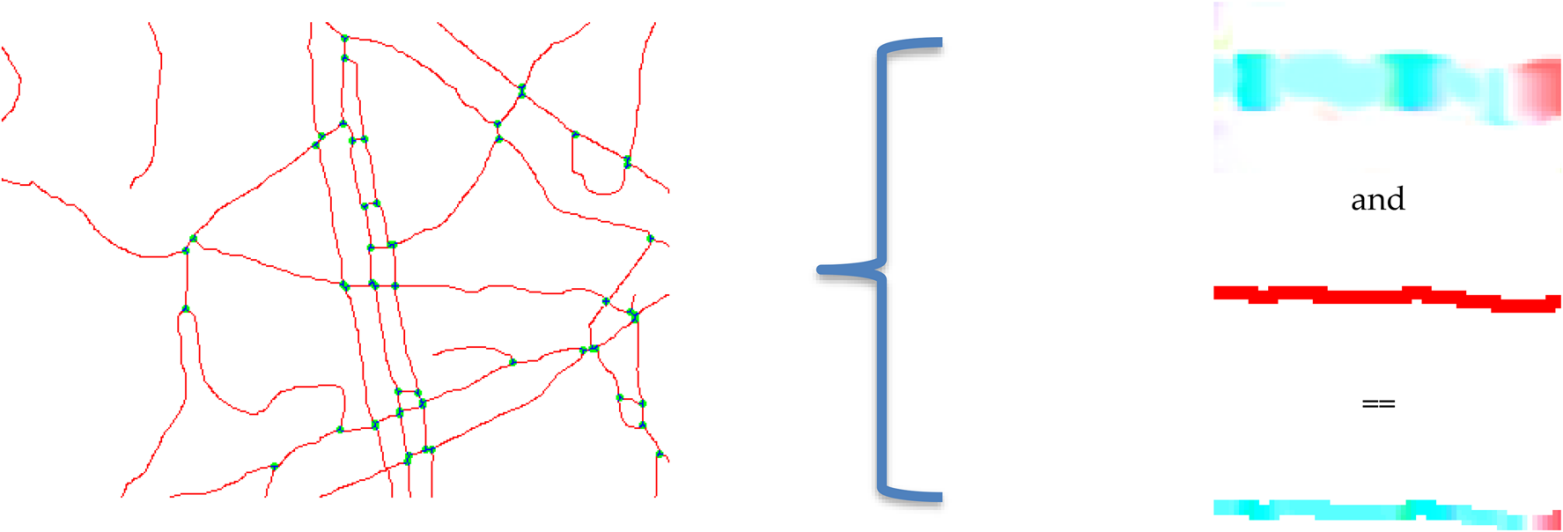
Vascular net skeleton with branch node points and extraction blood way with velocity estimation in branch.

The magnitudes of optical flow vectors are using for calculation of value of relative velocity of blood flow. But most strong adequacy of it collected on along the midline of the skeleton. Constriction of profile changing of optical flow along this line define distribution relative values of velocity for vessel branch (Fig. 9).

**Figure 10:**
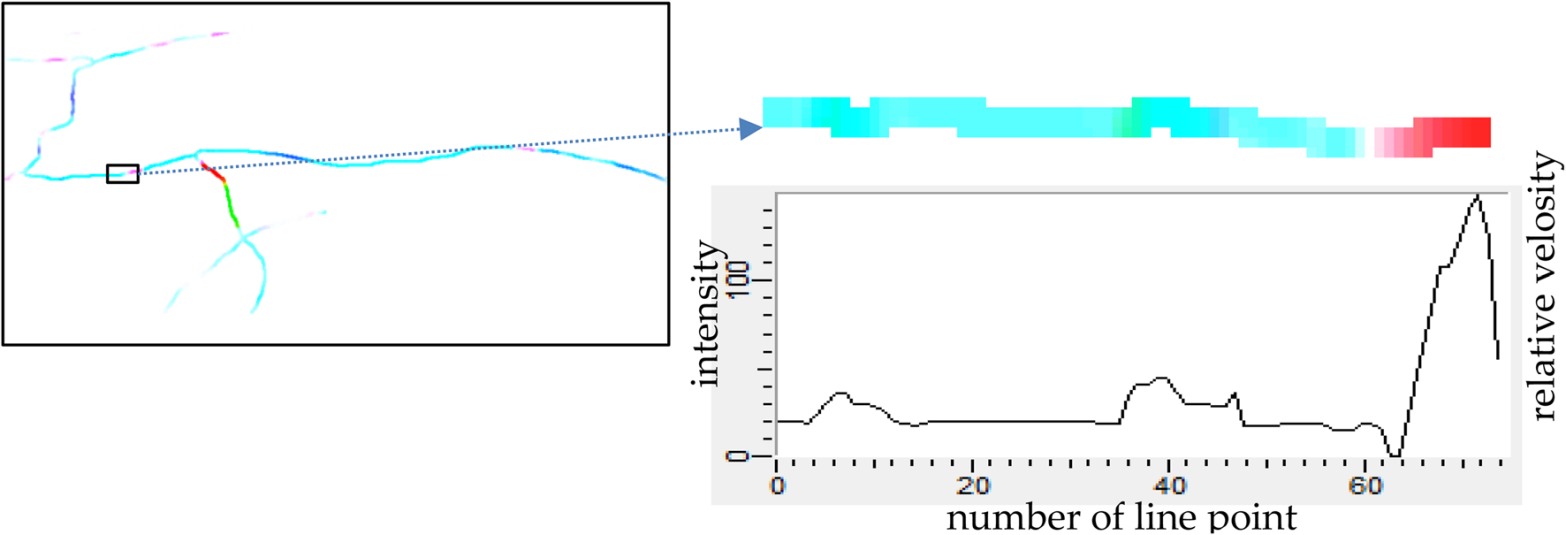
Optical flow in vessel branch and chart of relative velocity in vessel branch.

This profile represents a change in blood instantaneous linear speed for any point on the middle line of the vessel (Fig. 10).

### Institutional review board statement

The study did not require ethical approval.

### Informed consent

Informed consent was obtained from all subjects involved in the study.

## Results

Thus, as a result of calculating the optical flow, the motion vector for each pixel is determined. Knowing the time during which the position of the pixel has changed, we can calculate the instantaneous linear velocity of blood flow. Normally, in adults, the speed of propagation of a pulse wave in the vessels of the elastic type is 5–8 m/s, in the vessels of the muscular type—6–10 m/s. this is consistent with the normalized values of the optical flow magnitude.

The velocity of blood flow in the vessel and its diameter are used to record the instantaneous changes that occur in the vessel. Velocity determination based on optical flow and vessel width can be performed in parallel. The subsequent analysis of the results makes it possible to quantify changes in the linear velocity of blood flow in the vessels of healthy people when modeling the processes of hypercapnia and hyperoxia.

Problems associated with the discretization of time and space make it difficult to apply absolute values. The optical flow values were used to determine the instantaneous speed, which was measured in relative units^29^. The volumetric velocity of blood flow in the capillary depends on its width, it can be calculated by the next formula

9 $$Q = v\cdot S,$$

where $v$ is the linear velocity of blood flow, $Q$ is the volumetric velocity, $S$ is the cross-sectional area of the vessel.

Basic characteristics of segment of vascular net are width, length and blood flow speed. The length is detected for whole network or separate segments. But width and speed are detected only in one point. Therefore, for segment of vascular network such characteristics are calculated by averaging results for all selected points. Also, there is possibilities to construct of multiple adjacent blood vessel speed profiles. Other hemodynamic parameters can be calculated by topological transformation and description.

Thinning and segmentation of vessel reconstruct image to skeleton of pattern of vascular net that include branch points. These points are important topological elements where description of blood flow is changed. In result for vascular net it is possible to calculate common length, length of every segment of vascular branch, branchiness, compactness, and tortuosity, based on topological characteristics and such dynamical characteristics as velocity.

## Discussion

The proposed algorithm for measuring and monitoring the blood flow characteristics in the vessels allows the estimation of linear and volumetric blood speed and topological characteristics of the vascular net. The algorithm analyses the image sequentially and then builds optical flow maps for the video sequence. Dynamic characteristics of vessels are introduced and calculated. These characteristics determine changes in blood flow in natural eye vessel scenes.

The method was tested on the video sequence of blood vessels of the conjunctiva. The change in blood flow speed in vessels reflects the change in blood flow in the microcirculatory bed and other organs for normal and pathological conditions. The research and testing were realized by a high-resolution monochrome digital video camera Imperx Bobcat IGV-B1410M with a microscope lens with a focal length of 40 mm.

The linear speed of blood flow in a vessel with a diameter of 1.91 µm is 0.50379 relative units, corresponding to 5∙10–5 m/s. This result corresponds to the data obtained by the Doppler method.

## Conclusions

The proposed method allows spending research of the dynamic blood characteristics of the vascular net. The definition of instantaneous linear and volumetric speed for each vessel point describes eye diseases' properties at an early stage. The quantitative assessment of the cross-section area and linear and volumetric speed in vessels creates new properties of different pathologies, including the topology of vessel net. As AI can extrapolate the patterns in video data, further study on the blood flow pattern might allow us to identify some diseases in the future. Another future direction of research might throw light on studying specific types of blood vessels like retinal, improving existing study like^30^ that only study retinal images.

The developed methods of analyzing sclera video sequences and calculating the magnitude of the optical flow make it possible to quantify the change in the linear velocity of blood flow in them in healthy people when modelling hypercapnia and hyperoxia. The proposed method is a non-invasive method for the diagnosis of microcirculation disorders. It is used in medical institutions to study microcirculation in the vessels of the bulbar conjunctiva. In addition, according to several studies, the microcirculatory bed in the vessels of the bulbar conjunctiva can be used to judge the state of the microcirculatory bed of other organs and systems, for example, the brain, kidneys, and heart. Thus, having information about the state of the bulbar conjunctiva vessels makes it possible to assess the risk of developing hemodynamic disorders in other vessels.

## Data Availability

All experimental data necessary to confirm the results of this work are presented in the main article.
